# The potential of *Thymus vulgaris* aqueous extract to protect against delayed gastric emptying and colonic constipation in rats

**DOI:** 10.1039/c9ra02042j

**Published:** 2019-07-02

**Authors:** Kaïs Rtibi, Slimen Selmi, Dalanda Wannes, Mourad Jridi, Lamjed Marzouki, Hichem Sebai

**Affiliations:** Laboratory of Functional Physiology and Valorization of Bio-resources-Higher Institute of Biotechnology of Beja, University of Jendouba B. P. 382 9000 Beja Tunisia rtibikais@yahoo.fr +216 72 590 566 +216 97 479 135; Laboratory of Enzymatic Engineering and Microbiology, National School of Engineers of Sfax, University of Sfax B. P. 1173 3038 Sfax Tunisia

## Abstract

Thyme is a rich source of bioactive phytochemicals and it is frequently used in folk-medicine to treat gastroenteritis irritations. The current study was performed to examine the potential of *Thymus vulgaris* aqueous extract (TV-AE) to protect against delayed gastric emptying (DGE) and colonic constipation in rats. Stomach ulcer was caused by a single oral dose administration of indomethacin (INDO) (30 mg kg^−1^ of body weight). Constipation was induced following intravenous intoxication of rats with vinblastine (VINB) (2 mg kg^−1^ of body weight) for one week. The effect of TV-AE at two graduated doses (100 and 200 mg kg^−1^) on DGE, gastrointestinal transit (GIT) and constipated rats and biochemical parameters was estimated using phenol red, charcoal meal test and colorimetric methods, respectively. The phytochemical-profile of TV-AE was explored using high-performance liquid chromatography coupled with photodiode array detection and electrospray ionization-mass spectrometry (HPLC-PDA/ESI-MS). INDO and VINB caused a significant reduction in (*P* < 0.05) DGE and GIT and colonic motility dysfunction. TV-AE consumption remarkably (*P* < 0.05) attenuated the DGE (from 58.56% to 69.871%) and difficulty in evacuating stools (from 48.5 to 55.5 fecal pellets per rat) and balanced the GIT (65% to 71%). These GI-ameliorative effects were accompanied by reversed INDO/VINB-related oxidative changes, lipid-metabolism alteration and intracellular pathway moderation. HPLC-PDA/ESI-MS-analysis identified several chemical constituents including rosmarinic acid, quinic acid, luteolin-7-*o*-glucoside, protocatechuic acid and *trans*-cinnamic acid. Thus, TV-AE bioactive components may be used as medicinal substances to regulate/attenuate gastrointestinal–physiological activities and disturbances, which support its pharmacological use.

## Abstract 2

Thyme is a rich source of bioactive phytochemicals and it is frequently used in folk-medicine to treat gastroenteritis irritations.

## Graphical abstract

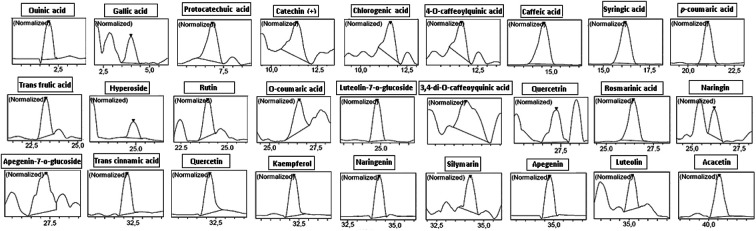

## 1. Introduction

Natural bioactive components are considered to be beneficial elements designed to improve human health.^1,2^ Plant products have the ability to transfer physiological benefits, including reduction of cancer risks, inflammatory states, cardiovascular diseases, diabetes and low-glycemic responses, neurodegenerative disorders, high blood cholesterol levels and reactive oxygen species and free radical overproduction.^3^

*Thymus vulgaris* L., an aromatic herbaceous plant, is a medicinal herb belonging to the Lamiaceae family. It is commonly cultivated and used in many countries mainly as a food seasoning and a source of essential oil that are used in the food industry, in perfumery as a cosmetic and for medical purposes.^4^ In traditional medicine, thyme is usually used in folk remedy in the prevention/treatment of a variety of disorders such as gastroenteric and broncho-pulmonary disruptions.^5^ As reported by several researchers, thyme contains numerous phenolic compounds, especially thymol and carvacrol, which are found in its essential oil. Additionally, in wild-thyme, many other abundant phenolic compounds have been found such as caffeic and rosmarinic acid derivatives.^6^

*Thymus vulgaris* extract has different pharmacological activities, which were confirmed *via in vivo* and *in vitro* experiments, including immunomodulatory activity, anti-inflammatory effect, antibacterial ability, anthelmintic action, anti-oxidant activity, anti-thrombin capacity and potent antihypertensive ability. The therapeutic potential of *Thymus vulgaris* is based on its content of aliphatic phenols, flavonoids, thymol, carvacrol, and eugenol, in addition to luteolin and saponins.^7–9^

Functional gastrointestinal disorders (FGIDs) are defined by the perturbation of gastrointestinal tract sections such as gastroenteritis, ulcerative colitis, functional constipation and delayed gastric emptying (DGE). FGIDs, also known as disturbances of the gut–brain interaction, contain a number of separate idiopathic disruptions, which alter different regions of the GI-tract and involve visceral motility disorders related to an oxidative stress installation.^10,11^ Excessive ROS generation may contribute to cell injuries by lipid-protein-DNA oxidation in various tissues.^12^ In the GI tract, the cellular redox potential may even determine the fate of the differentiating cells. Wnt/β-catenin and notch signaling pathways are the major determinants of cell commitment for specialization. There may be a direct association between NADPH oxidase 1 (NOX-1)-dependent ROS fabrication, differential provocation of Wnt/β-catenin or notch signaling pathways, and intestinal proliferation. Modifications in the redox capacities of GSH/GSSG and cysteine/cystine (Cys/CySS) have been integrated with intestinal phenotypic transitions. The reducing abilities may favor cell proliferation, but oxidizing efficiencies can lead to cell growth reduction or inhibition.^13,14^

There are no published reports in the literature about the effect of the extract of *T. vulgaris* on gastric emptying and functional constipation. Based on these considerations, this study was designed to characterize the various TV-AE phenolic components and to investigate the potentiality of TV-AE to protect the GI tract against gastric delayed emptying and colonic constipation in rats.

## 2. Materials and methods

### 2.1. Chemicals

Clonidine, red phenol, NaCl, NaOH, charcoal meal, gum arabic, and haematoxylin/eosin were purchased from Sigma Chemical Co. (Sigma-Aldrich GmbH, Steinheim, Germany). Indomethacin and vinblastine were purchased from a local central pharmacy. All other reagents used were of analytical grade.

### 2.2. TV-AE preparation

*T. vulgaris* leaves were collected from the region of Jendouba (North-West of Tunisia) during the season of spring (2017). Voucher specimens were identified and authenticated by a taxonomist in the Higher Institute of Biotechnology of Beja Tunisia and deposited at the herbarium in the Faculty of Sciences, University of El Manar, Tunisia. The leaves were dried at 40 °C in air, ground, and extracted with bi-distilled water. The obtained leaf extracts were dehydrated at the same temperature under vacuum and finally freeze dried.

### 2.3. TV-AE bioactive compound identification by liquid chromatography-high-resolution electrospray ionization mass spectrometry (LC-HRESIMS) assay

100 mg of TV-AE was dissolved in 100 mL of 10% methanol, filtered and then 1 mL was added to LC-MS vials. A reverse-phase column (Pursuit XRs ULTRA 2.8, C18, 100 × 2 mm, Agilent Technologies, UK) was used to perform HPLC examinations. 20 mL of the sample was injected at a column temperature set at 30 °C. The mobile phase consisted of 0.1% formic acid in water (A) and 0.1% formic acid in methanol (B). A gradient program was used for separation at a flow rate of 1 mL min^−1^. The mobile phase consisted of an initial composition of 100% solvent A, with a gradient of 100% solvent B over 20 min, held at 100% solvent B for 5 min and 100% solvent A for 25 min. The drying gas flow rate was 1 mL min^−1^ at 320 °C. MS was operated in the positive ion mode in the mass range of 100–2000 *m*/*z*. High-resolution mass spectral data was obtained on a Thermo Instruments ESI-MS system (LTQ XL/LTQ Orbitrap Discovery, UK) connected to a Thermo Instruments HPLC system (Accela PDA Detector, Accela PDA Autosampler and Accela Pump).^15^

### 2.4. Animals

Male Wistar rats (180 and 220 g) were used at 13 weeks of age. The animals were obtained from the Society of Pharmaceutical Industries of Tunisia (SIPHAT, Ben-Arours, Tunisia), and isolated into different groups and acclimatized for two weeks with a standard pellet diet (standard pellet diet Badr-Utique-TN) and water ad libitum (22 ± 2 °C; 12 h dark per light cycle). All animal procedures were performed in accordance with the Guidelines for Care and Use of Laboratory Animals of Tunis University and approved by the Animal Ethics Committee of National Institute of Health.^16^

### 2.5. Experimental design

#### 2.5.1. Acute DGE and CON inductions in rats

Acute UG was caused in the animals according to the procedure described by Oluwabunmi and Abiola.^17^ Briefly, the rats were administered with a single oral dose of indomethacin (30 mg kg^−1^ body weight). The animals were deprived of food for 24 h but had free access to water prior to ulcer induction. Various degrees of ulceration manifested 4 h after indomethacin administration. Acute UG diagnosis was evaluated by different clinical signs, such as gastric juice volume, ulcer score and gastric pH. Colonic alterations were caused *in vivo* according to the method elucidated by Rtibi *et al.*^18^ Briefly, animals were intoxicated with intravenous injection of vinblastine (2 mg kg^−1^ of body weight in a saline solution) for 7 days. Diagnosis of VINB injuries was confirmed by several signs, such as fecal pellet, wet fecal weight, dry fecal weight and water content.

#### 2.5.2. TV-AE and drug actions on GIT in healthy and treated rats

This manipulation was carried out according to our previous studies using the charcoal meal test.^19^ Specifically, after 2 h of animal treatment with TV-AE at two doses (100 and 200 mg kg^−1^) or the standard molecule (1 mg kg^−1^), the colitis rats were provided the standard charcoal meal (10% charcoal in 5% gum arabic) by oral administration. After 30 min, the animals were anesthetized and their small bowel was excised. The distance travelled by the charcoal meal suspension was measured and expressed as a percentage of the total length. The peristaltic index was calculated using the following formula:Peristaltic index = (distance travelled by charcoal meal/length of small-intestine) × 100% of inhibition = mean distance travelled by the control − mean distance travelled by the test group/mean distance travelled by the control.

#### 2.5.3. INDO and TV-AE effects on gastric emptying process

To assess the INDO/TV-AE actions on the gastric-emptying process, the red phenol method was used. In this context, the animals were divided into different groups of seven and administered 1 h before with the test meal (50 mg phenol red in 100 mL aqueous methyl cellulose) as follows: group 1 served as the control group (CONT) and received 1 mL of physiological solution (NaCl, 0.9%, p.o.). Groups 2 and 3 were treated only with the extract. Group 4 served as the INDO group without extract and drug. Groups 5 and 6 of INDO rats were pre-treated with two doses of TV-AE (100 and 200 mg kg^−1^, b.w. p.o.).

After 1 h, the animals were anesthetized and euthanized. Their stomach and its contents were combined with 100 mL of 0.1 N NaOH. The supernatants were mixed with 4 mL of 0.5 N NaOH and the absorbance of the samples was read at 560 nm. Phenol red collected immediately from stomach organs after test meal administration was used as the standard (0% of GE).^20^

GE (%) was calculated according to the following formula:Gastric emptying rate (%) = (1 − absorbance of treated/absorbance of standard) × 100.

#### 2.5.4. VINB and TV-AE effects on colonic motor activity

The animals were randomly divided into four groups. Healthy animals or the control group (CONT) received a physiological solution at a dose of 5 mL kg^−1^ (*n* = 7). The VINB group was treated intravenously only with VINB (2 mg kg^−1^ b.w.) dissolved in a saline solution once per day for one week (*n* = 7). Finally, the two other groups were treated by VINB in combination with TV-AE at two doses of 100 mg kg^−1^ and 200 mg kg^−1^ (*n* = 7). On day 5, the faecal pellet weights (wet and dry weights) of the rats were measured for 24 h and the water levels were determined by the difference between the weights before and after drying.^18^

#### 2.5.5. Oxidative and antioxidative marker assessment in rats

Gastric and colonic mucosal tissues were scraped and then placed in a phosphate buffered saline (PBS) solution, homogenized and centrifuged for 15 min at 9000 × *g*. The obtained aliquots were stored at −80 °C.

Lipid oxidation caused by ROS and free radicals in the UG/UC rats led to MDA production, which was estimated with double heating technique adapted by Draper *et al.*^21^ The expression of the results was reported as the mean of MDA nmol mg^−1^ of proteins. Thiol group (–SH) content was determined using Ellman's procedure^22^ and the data was expressed as nmol of –SH per mg of proteins. Carbonyl residue generation after protein oxidation was examined using the method by Levine *et al.*^23^ The results were expressed as μmol carbonyl residue per mg protein.

Superoxide dismutase action was determined using the nicotinamide adenine dinucleotide (reduced) phenazine methosulphate nitroblue tetrazolium reaction system method.^24^ The results were calculated and expressed in units of SOD per mg proteins. Catalase (CAT) activity was estimated using the procedure by Aebi^25^ and the results were expressed as nmol min^−1^ mg^−1^ protein. GPx activity was analysed using the Flohé and Günzler design^26^ and the data was expressed as nmol GSH min^−1^ mg^−1^ protein.

#### 2.5.6. Lipid indicators and moderating intracellular pathways assessment in rats

After blood centrifugation at 3000 × *g* for 10 min, the lipid marker (triglycerides, total cholesterol, and high-density cholesterol) contents were measured in the as-obtained serum using commercially available kits purchased from Biomaghreb, Tunisia.

Gastric and colonic mucosal H_2_O_2_ levels were determined according to the method by Dingeon *et al.*^27^ and the data was expressed as μmol of H_2_O_2_ per mg protein. Intracellular mediators (calcium and non-haem iron) levels were measured using commercial kits purchased from Biomaghreb, Tunisia.^28,29^ The results were expressed as nmol of these parameters per mg protein.

### 2.6. Statistical analysis

A one-way analysis of variance test was used to determine the significance between the different groups of all animals. Statistical analyses were calculated using StatView statistical software. The data is representative of six to eight distinct observations. Differences were stated as mean ± SEM and designated significant when the values of *p* were <0.05.

## 3. Results and discussion

### 3.1. Phytochemical composition

**Fig. 1:**
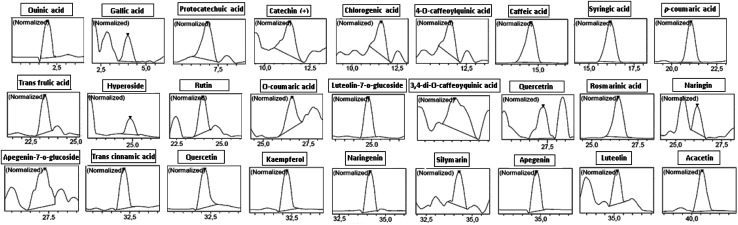
Chromatographic profiles and characterization of phenolic compounds in TV-AE.

It has been reported that thyme has been considered for long time as a good source of phenolic compounds.^30^ In this respect, HPLC-PDA/ESI-MS analysis revealed the presence of 27 phenolic-compounds in TV-AE, including rosmarinic acid, quinic acid, luteolin-7-*o*-glucoside, protocatechuic acid, trans cinnamic acid, caffeic acid, naringenin, kaempferol, quercetin, apigenin, rutin, 3,4-di-*O*-caffeoylquinic acid, syringic acid, *p*-coumaric acid, naringin, acacetin, hyperoside (quercetin-3-*o*-galactoside), quercetrin (quercetin-3-*o*-rhamonosid), gallic acid, *trans* ferulic acid, apigenin-7-*o*-glucoside, 4-*O*-caffeoylquinic acid, chlorogenic acid, silymarin, catechin(+), luteolin and *o*-coumaric acid (Fig. 1 and Table 1). Moreover, according to previous research, the phenolic profile of thyme obtained with HPLC-PDA/ESI-MS analysis showed diverse phenolic compounds such as rosmarinic acid, quinic acid, luteolin-7-*O*-glucoside, protocatechuic acid, *trans* cinnamic acid, caffeic acid, naringenin, kaempferol, quercetin, apigenin, rutin, 3,4-di-*O*-caffeoylquinic acid, syringic acid, *p*-coumaric acid, naringin, acacetin, hyperoside (quercetin-3-*o*-galactoside), quercetrin (quercetin-3-*o*-rhamonosid), gallic acid, *trans* ferulic acid, apigenin-7-*o*-glucoside, 4-*O*-caffeoylquinic acid, chlorogenic acid, silymarin, catechin(+), luteolin and *o*-coumaric acid.^31^ Importantly, many researchers (*in vitro*, *in silico* and *in vivo*) have proven the structure–activity relationship of bioactive compounds such as phenolic acids and flavonoids with their biological properties. In this respect, the bioactivity of these compounds depends mostly on the number of hydroxyl groups linked to the aromatic ring and their relative orientation in the molecular structure.^32^

**Table 1:** High-resolution liquid chromatography/electrospray ionization (LC-HRESIMS) TV-AE component identification

Name^a^	Molecular formula	PubChem CID	[M]^−^H^b^*m*/*z*	Retention time	Concentration (ppm)
Quinic acid	C_7_H_12_O_6_	6508	191.00	1.978	364.647
Gallic acid	C_7_H_6_O_5_	370	169.00	3.989	6.688
Protocatechuic acid	C_7_H_6_O_4_	72	153.00	6.890	46.790
Catechin(+)	C_15_H_14_O_6_	73 160	289.00	11.382	1.311
Chlorogenic acid	C_16_H_18_O_9_	1 794 427	353.00	11.615	2.493
4-*O*-Caffeoylquinic acid	C_16_H_18_O_9_	5 281 780	353.00	11.612	2.558
Caffeic acid	C_9_H_8_O_4_	689 043	179.00	14.603	37.468
Syringic acid	C_9_H_10_O_5_	10 742	197.00	16.190	24.471
*p*-Coumaric acid	C_9_H_8_O_3_	637 542	163.00	21.061	17.003
*trans* Ferulic acid	C_10_H_10_O_4_	445 858	193.00	23.276	3.501
Hyperoside (quercetin-3-*o*-galactoside)	C_21_H_20_O_12_	5 281 643	463.00	24.864	10.149
Rutin	C_27_H_30_O_16_	5 280 805	609.00	23.941	28.653
*o*-Coumaric acid	C_9_H_8_O_3_	637 540	163.00	26.598	1.258
Luteolin-7-*o*-glucoside	C_21_H_20_O_11_	5 280 637	447.00	24.800	46.983
3,4-Di-*O*-caffeoylquinic acid	C_25_H_24_O_12_	5 281 780	515.00	25.146	24.494
Quercetrin (quercetin-3-*o*-rhamnoside)	C_21_H_20_O_11_	15 939 939	447.00	27.267	7.219
Rosmarinic acid	C_18_H_16_O_8_	5 315 615	359.00	26.434	5066.278
Naringin	C_27_H_32_O_14_	442 428	579.00	26.168	11.984
Apigenin-7-*o*-glucoside	C_21_H_20_O_10_	45 933 926	431.00	27.291	3.393
*trans* Cinnamic acid	C_9_H_8_O_2_	444 539	147.00	32.184	41.109
Quercetin	C_15_H_10_O_7_	5 280 343	301.00	32.175	35.730
Kampherol	C_15_H_10_O_6_	5 280 863	285.00	32.218	35.972
Naringenin	C_15_H_12_O_5_	439 246	271.00	34.164	37.425
Silymarin	C_25_H_22_O_10_	31 553	481.00	34.290	1.547
Apigenin	C_15_H_10_O_5_	5 280 443	269.00	34.768	35.701
Luteolin	C_15_H_10_O_6_	5 280 445	285.00	35.175	1.270
Acacetin	C_16_H_12_O_5_	5 280 442	283.00	40.566	11.362

a The compounds are suggested according to the dictionary of natural products and the characteristic fragmentation pattern.

b The formulas were deduced from the quasi molecular ion peak [M + H]^+^.

### 3.2. TV-AE consumption accelerates GIT in healthy and constipated rats

Chemotherapy-induced constipation (CIC) is a persistent problem in the treatment of different types of cancer, and is among the primary contributors to dose decreases, delays and inefficient treatment.^33^ In the VINB rats, the overall gastrointestinal motor activity analysed by the progression of the test meal through the small-bowel was significantly (*P* < 0.05) reduced (65.12 ± 1.66%) compared to those in the healthy animals (70.65 ± 3.63%). However, TV-AE administration at both doses resulted in a significant (*P* < 0.05) improvement in gastrointestinal transit in both the healthy (77.92 ± 3.45 and 83.74 ± 2.88%, respectively) and VINB rats (68.90 ± 2.18 and 71.33 ± 3.46%, respectively) (Table 2). Furthermore, the rats receiving VINB showed several signs, including severe difficulty in evacuating stools, which was observed in the CON model compared with the healthy group. This effect was confirmed by the faecal parameter disorders noticed in the VINB group at day 5, including faecal pellets and water content diminution (48.64 ± 3.21 fecal pellets and 27.66 ± 2.63% water content) in comparison with the control-group (56.23 ± 3.00 fecal pellets and 39.08 ± 3.10% water content) (Table 3). These alterations were combined with histopathological-injuries,^34,35^ which provoked loss of fluidity/permeability. These eruptions were accompanied with equilibrium disruption of the absorption/secretion of water, electrolytes and nutrients, which led to diverse variations in the gastrointestinal physiological functions, such as GIT. TV-AE administration removed these disruptions, and thus allowed the construction of the mucosa, as shown by the histological study and the facilitation of intestinal and colonic contents advancement.^36,37^

**Table 2:** VINB/TV-AE actions on gastrointestinal motility in healthy and intoxicated rats^a^

Group	Dose	Gastro-intestinal motility (%)	Reduction activity (%)	Reinforcement (%)
Control (NaCl)	(5 mL kg^−1^)	70.65 ± 3.63	—	—
Clonidine	(1 mg kg^−1^)	28.23 ± 1.77*	60.04	—
VINB	(2 mg kg^−1^)	65.12 ± 1.66*	07.82	—
TV-AE alone	(100 mg kg^−1^)	77.92 ± 3.45*^#^	—	10.29
	(200 mg kg^−1^)	83.74 ± 2.88*^#^	—	18.52
VINB/TV-AE	(100 mg kg^−1^)	68.90 ± 2.18^#^	—	5.80
	(200 mg kg^−1^)	71.33 ± 3.46^#^	—	09.53

a Rats were intoxicated with VINB (2 mg kg^−1^) for one week. Then, the animals were treated with two increasing doses of TV-AE or reference molecule (clonidine, 1 mg kg^−1^). 2 h later, the rats were given the standard charcoal meal (10% charcoal in 5% gum arabic) or vehicle (NaCl, 0.9%). Data are expressed as mean ± standard error (*n* = 7); *P < 0.05 *versus* control group, ^#^*P* < 0.05 *versus* VINB-group.

**Table 3:** Identification of parameters following acute drug disorders and TV-AE effects^a^

Rat group	Fecal parameters on day 5 (collection for 24 h)				Gastric mucosa injury indicators		
	Fecal pellet (n)	Wet weight (g/24 h/rat)	Dry weight (g/24 h/rat)	Water content (%)	Ulcer index	Gastric juice volume (mL)	Gastric juice pH
CONT (NaCl)	56.23 ± 3.00	6.77 ± 4.64	3.21 ± 0.33	39.08 ± 3.10	0.23 ± 0.04	2.04 ± 0.14	3.46 ± 0.09
VINB (2 mg kg^−1^)	48.64 ± 3.21*	4.23 ± 0.21*	2.22 ± 0.70*	27.66 ± 2.63*	—	—	—
INDO (30 mg kg^−1^)	—	—	—	—	19.77 ± 1.56	4.44 ± 0.16	2.31 ± 0.06
TV-AE (100 mg kg^−1^)	51.11 ± 2.90*	4.93 ± 0.55*	2.97 ± 0.61*	31.17 ± 3.55*	10.64 ± 1.20*	2.62 ± 0.37*^#^	2.87 ± 0.12*^#^
TV-AE (200 mg kg^−1^)	55.50 ± 1.72^#^	5.64 ± 0.81^#^	3.24 ± 0.10^#^	37.44 ± 2.85^#^	3.00 ± 0.06*	2.12 ± 0.22^#^	3.72 ± 0.10^#^

a VINB/INDO alterations reflected by indicated parameters and assessed in healthy rats and rats treated with VINB (2 mg kg^−1^) or INDO (30 mg kg^−1^). Data are expressed as mean ± standard error (*n* = 7); *P < 0.05 *versus* control group, ^#^*P* < 0.05 *versus* VINB/INDO groups.

Actually, the exact mechanisms of the action of CIC remain unclear, but are believed to result from the association of intersecting processes, including GI dysmotility, secretory dysfunction, inflammation and alterations in GI innervation.^33^

TV-AE may contain many bioactive compounds that behave as purgatives/stimulate, which activates the gut musculature by acting on the nerve endings of the colon and causing movements that promote defecation. They also act on the walls of the intestine and increase mineral/liquid production, while lessening the absorption of sodium/chlorine.^38^

In addition, interstitial cells of Cajal (ICCs) appear to play crucial roles in the physiological GI motility modulation and act as the pacemaker of gastrointestinal movement. In this respect, we think that TV-AE can depolarize the membrane potentials of ICCs. This excitation may lead to smooth muscle cells *via* a gap junction. These last cells may respond to this depolarization with the activation of voltage-dependent Ca^2+^ channels.^39^ Therefore, this action of pacemaker potential depolarization may produce intensification of the intestinal motility rate. For this point, many recent research showed that diverse traditional herbal medicines such as *Schisandra chinensis*,^40^ Ge-Gen-Tang,^41^*Liriope platyphylla*^42^ and *Citrus unshiu*^43^ demonstrated elicit excitatory/inhibitory effects on the ICC pacemaker activity, which supports the notion that ICCs are critical in the control of smooth muscle motility in the GI tract.

Direct assessments of calcium currents in single smooth muscle cells by the whole cell voltage clamp methods in the rat and mouse colons showed a 50–70% decrease in Ca^2+^ ion flux after an inflammatory response. Akbarali *et al.*^44^ established that in addition to the down-regulation of Ca^2+^ currents, the response to the ATP-sensitive K^+^ channel agonist lemakalim improved in the inflamed smooth muscle cells. Likewise, colonic irritation results in contracted contractions of colonic muscle strips in both human/animal models of colitis to depolarizing K^+^ solutions. In particular, recent studies indicated that transient receptor potential vanilloid-4 (TRPV4) activation in the GI tract induces intracellular calcium accumulation, chemokine release enhancement, and colitis reinforcement. In contrast, anti-inflammatory compounds significantly diminished TRPV4-expression in the upper-intestine.^45,46^

### 3.3. TV-AE consumption facilitates the emptying process in healthy and INDO group

NSAIDs stimulate gastric ulcers *via* the suppression of prostaglandins, which are cytoprotective constituents to the gastric mucosal tissues, specifically due to the COX pathway disturbance of arachidonic acid metabolism, resulting in the massive generation of leukotrienes and other factors such as that of the 5-lipoxygenase enzyme metabolic pathway.^47^

**Fig. 2:**
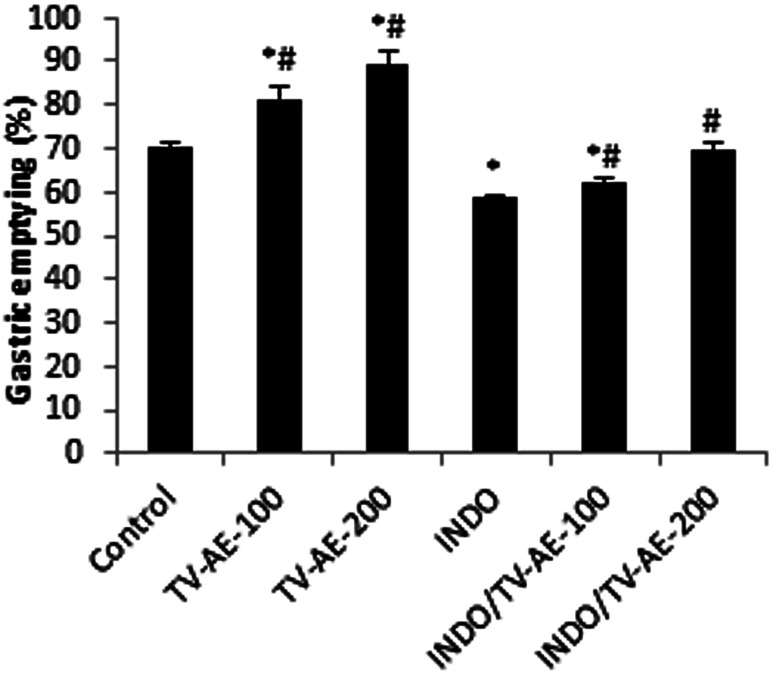
Effect of TV-AE on INDO-induced delayed gastric emptying. Animals were treated 1 h prior to the test meal (50 mg phenol red in 100 mL aqueous methyl cellulose) with two doses of TV-AE (100 and 200 mg kg^−1^). Results are expressed as mean ± SEM; *n* = 7 in each group. Data was analyzed by Statview ANOVA. *P < 0.05 compared to control group, ^#^*P* < 0.05 compared to INDO group.

INDO administration at a dose of 30 mg kg^−1^ produced acute ulcerative gastritis in the animals. This damage was manifested by an increase in the ulcer index and gastric juice volume and a significant reduction in pH. These disruptive actions were accompanied with a delayed gastric emptying effect.^17^ TV-AE at both concentrations induced a significant (*P* < 0.05) acceleration of normal gastric emptying (81.52 ± 2.67% and 89.05 ± 3.44%, respectively) when the results are compared to that found in the healthy animals (70.25 ± 1.45%). In the ulcerative gastritis situation, the acquired data showed that the gastric emptying time analysed by the red phenol method strongly (*P* < 0.05) decreased in the gastric ulcer situation (58.56 ± 0.78%). In contrast, comparatively with the ulcerative gastritis group, TV-AE pre-treatment attenuated UG and remarkably accelerated (*P* < 0.05) the gastric emptying time (62.40 ± 0.99% and 69.87 ± 1.66%, respectively) (Fig. 2).

Natural small molecules produced from medicinal plants have been used for a long time to treat and prevent diverse ailments. Recently, the use of natural medicine in the treatment of various pathologies like peptic ulcer is an absolute requirement. Therefore, an alternative advance in recent days is the research of phytomedicaments serving as a tool in the prevention of peptic ulcers.^48^ Several phyto-components such as flavonoids, tannins, terpenoids, and saponin have been reported in distinct anti-ulcer findings as possible gastro-protective agents. Rosmarinic acid is known to be facilely absorbed from the gastrointestinal tract into the blood. It activates prostaglandin E2 generation, diminishes leukotriene B4 production in human leucocytes, and inhibits the complement system. It is also proven that caffeic acid and derivatives such as rosmarinic acid have therapeutic potential in the treatment/prevention of spasmogenic disruptions, peptic ulcers, and inflammatory disorders.^49^

### 3.4. TV-AE consumption relieves and ameliorates oxidative and antioxidative balance

**Fig. 3:**
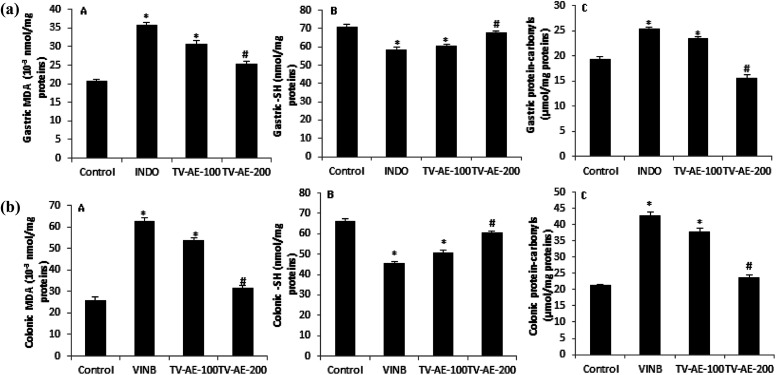
(a and b) Acute effect of TV-AE (100 and 200 mg kg^−1^, respectively) on INDO/VINB-induced changes in gastric and colonic mucosal MDA, SH group and protein carbonyls levels. *P < 0.05 compared to control group, ^#^*P* < 0.05 compared to INDO and VINB-groups. Values are means ± SEM (*n* = 7).

**Fig. 4:**
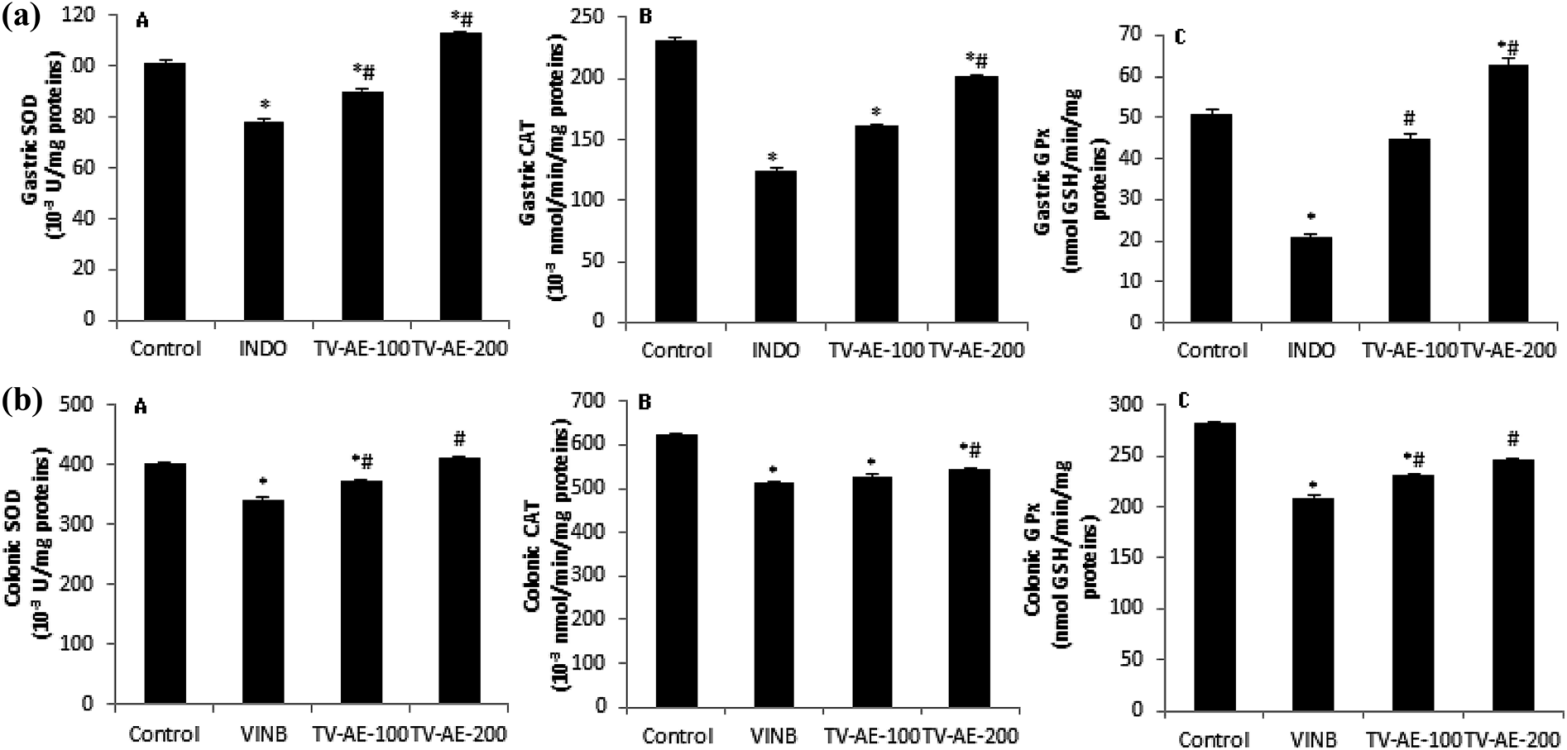
(a and b) Acute effect of TV-AE (100 and 200 mg kg^−1^, respectively) on INDO/VINB-induced depletion in gastric and colonic mucosal enzymatic antioxidant activities. *P < 0.05 compared to control-group, ^#^*P* < 0.05 compared to INDO and VINB-groups. Values are means ± SEM (*n* = 7).

In both INDO/VINB groups, the obtained results showed an oxidative stress installation, which is related with an enzymatic antioxidant disruption following a massive ROS/free radical generation. These injuries were confirmed by the increase in MDA production and carbonyl protein formation in the UG/UC conditions, and associated with the depletion of enzyme activities, such as SOD, CAT and GPx. However, TV-AE consumption at both different doses (100 and 200 mg kg^−1^) relieved oxidative damage and ameliorated/stimulated the antioxidant profile (Fig. 3 and 4).

A recent study clearly exhibited the participation of oxidative stress/inflammation in gastrointestinal problems such as ulcers and constipation.^50^ This contribution was checked by the augmentation of intracellular ROS production, which induces damage to lipids, proteins and DNA and inflammatory indicators. Iron catalyzes hydroxyl radical-mediated oxidative injury through its implication in the Fenton pathway, which leads to the reduction of this element in oxidative stress condition. Many authors showed that the phenolic compounds of thyme extract have greater antioxidant activity compared to α-tocophenol and butylated-hydroxylanisole. Rosmarinic acid (RA), the most important component of the thyme extract, has strong antioxidative activity since it acts as a free radical scavenger.^51^ Further research suggested other possible mechanisms for the protection of RA against lipid peroxidation, including the scavenging effect of numerous radical species, interaction with peroxyl-radicals and partitioning into the LDL particle and terminating chain reactions of oxidative degradation of lipids by scavenging lipid radicals.^52^ An alternative hypothesis is that antioxidant supplements modulate endogenous mechanisms that either decrease ROS production or increase the enzymes activities that decompose ROS.^53^

### 3.5. TV-AE consumption improves lipid metabolisms/intracellular mediators disruptions in intoxicated rats by drugs

To investigate the cell level damage and modifications in lipid metabolism, we studied the effect of drugs on lipidemia indicators as well as intracellular mediators. Accordingly, we observed a significant (*P* < 0.05) increase in H_2_O_2_ production in both eruptions, which was accompanied by a depletion of calcium and free iron. TG, TC and HDL cholesterol were significantly (*P* < 0.05) altered in the GU condition. However, the bioactive compounds and mineral constituents in TV-AE resulted in protection against all these disruptions (Table 4).

**Table 4:** VINB/INDO/TV-AE actions on intracellular mediator disturbances in healthy and treated rats^a^

Group	Dose	H_2_O_2_ (μmol mg^−1^ protein)	Free iron (nmol mg^−1^ protein)	Calcium (10^−3^ nmol mg^−1^ protein)
CONT (NaCl)	(5 mL kg^−1^)	09.05 ± 0.33	11.34 ± 0.06	33.67 ± 3.57
VINB	(2 mg kg^−1^)	25.77 ± 2.13*	7.04 ± 0.17*	19.47 ± 2.07*
VINB/TV-AE	(100 mg kg^−1^)	24.70 ± 2.11*^#^	9.24 ± 0.21	22.99 ± 0.84*
	(200 mg kg^−1^)	11.98 ± 1.22^#^	10.24 ± 0.06^#^	28.90 ± 1.34^#^
INDO	(30 mg kg^−1^)	37.33 ± 2.29*	04.28 ± 0.07*	21.38 ± 1.01*
INDO/TV-AE	(100 mg kg^−1^)	19.15 ± 1.37*^#^	07.52 ± 0.20*^#^	27.10 ± 0.74^#^
	(200 mg kg^−1^)	12.65 ± 0.93^#^	09.35 ± 0.13^#^	30.80 ± 2.21^#^

a VINB/INDO/TV-AE effects were reflected by intracellular mediators assessed in healthy rats and rats treated with VINB (2 mg kg^−1^) or INDO (30 mg kg^−1^). Results are expressed as mean ± SEM; *n* = 7 in each group. Data was analyzed by Statview ANOVA. Data are expressed as mean ± standard error (*n* = 7); *P < 0.05 *versus* control group, ^#^*P* < 0.05 *versus* VINB/INDO-groups.

Previous studies demonstrated that RA exhibited substantial H_2_O_2_ scavenging action and inhibited H_2_O_2_-induced intracellular ROS generation.^54^ The data also showed that the lipid components such as triglyceride, total cholesterol, and HDL cholesterol contents were remarkably different among the major groups (Table 5). A recent study reported that many bioactive components such as gallic acid, catechin, and epicatechin have cholesterol-lowering activities by reducing pancreatic cholesterol esterase activity, binding of bile acids, and inhibiting solubility of cholesterol in micelles, which may result in delayed cholesterol absorption. These flavonoids may also reduce cholesterol absorption *via* interaction with cholesterol carriers and transporters across the brush border membrane in rats.^55^ Abdulkarimi *et al.*^4^ showed that thyme extract consumption decreased the plasma triglyceride, total cholesterol, LDL-c and VLDL-c, which in turn lowered the liver and abdominal lipids, and reduced the proportional liver and abdominal fat weights. These actions were exerted by the lower activity of the HMG-CoA reductase enzyme, diminished fat absorption from the gastrointestinal tract or the lipid catabolism for the gluconeogenesis process.

**Table 5:** VINB/INDO/TV-AE actions on serum lipid metabolism disruptions in healthy and treated rats^a^

Group	Dose	Triacylglycerol (mg dL^−1^)	Total cholesterol (mg dL^−1^)	HDL-cholesterol (mg dL^−1^)
CONT (NaCl)	(5 mL kg^−1^)	80.00 ± 5.28	61.34 ± 4.51	32.13 ± 2.45
VINB	(2 mg kg^−1^)	78.40 ± 6.81	62.03 ± 5.12	30.40 ± 2.30
VINB/TV-AE	(100 mg kg^−1^)	77.37 ± 4.02	61.45 ± 3.59	33.12 ± 0.93
	(200 mg kg^−1^)	79.80 ± 2.87	60.23 ± 5.10	26.06 ± 1.21*^#^
INDO	(30 mg kg^−1^)	67.12 ± 3.50*	78.22 ± 2.15*	35.46 ± 1.57
INDO/TV-AE	(100 mg kg^−1^)	70.59 ± 4.21*	75.68 ± 3.17*	40.16 ± 3.55*^#^
	(200 mg kg^−1^)	81.35 ± 5.32^#^	63.27 ± 4.22^#^	44.86 ± 2.22*^#^

a VINB/INDO/TV-AE effects were reflected by serum lipid metabolism assessed in healthy rats and rats treated with VINB (2 mg kg^−1^) or INDO (30 mg kg^−1^). Results are expressed as mean ± SEM; *n* = 7 in each group. Data was analyzed by Statview ANOVA. Data are expressed as mean ± standard error (*n* = 7); *P < 0.05 *versus* control group, ^#^*P* < 0.05 *versus* VINB/INDO-groups.

## 4. Conclusion

The current study demonstrated that TV-AE represents a source of bioactive functional components and may contribute an improvement in gastrointestinal physiological actions and their associated disorders in rats *via* various mechanisms. Consequently, this investigation supports the therapeutic potential and strong possibility of TV-AE as a functional food in phytomedicine, which should be further explored in clinical studies.

## Financial disclosures

None declared.

## Conflicts of interest

Only the authors are responsible for the content of this paper.

## Abbreviations

CAT: Catalase
CIC: Chemotherapy induced constipation
CON: Constipation
COX: Cyclooxygenase
DGE: Delayed gastric emptying
FGID: Functional-gastrointestinal-disorders
GE: Gastric emptying
GET: Gastric emptying time
GIT: Gastrointestinal transit
GPx: Glutathione peroxidase
GU: Gastric ulcer
HDL: High density lipoproteins
HPLC-PDA/ESI-MS: Liquid chromatography-photodiode-array-mass spectrometry
ICCs: Interstitial cells of Cajal
INDO: Indomethacin
MDA: Malondialdehyde
NOX-1: NADPH-oxidase 1
NSAIDs: Nonsteroidal anti-inflammatory drugs
ROS: Reactive oxygen species
SOD: Superoxide dismutase
TC: Total cholesterol
TG: Triglycerides
TRPV4: Transient receptor potential vanilloid-4
TV-AE: *Thymus vulgaris* aqueous extract
UC: Ulcerative colitis
UG: Ulcerative gastritis
VINB: Vinblastine

## Supplementary Material
